# Guided Alveolar Ridge Preservation (G-ARP) Using a Cortical Lamina: A Pilot Randomized Controlled Trial

**DOI:** 10.3390/dj14040193

**Published:** 2026-03-24

**Authors:** Giacomo Mainetti, Franco Bengazi, Tomaso Mainetti, Karol Alí Apaza Alccayhuaman, Andrea Grassi, Eddy Troya Borges, Daniele Botticelli

**Affiliations:** 1 ARDEC Academy, 47923 Rimini, Italy; giacomo.mainetti@hotmail.it (G.M.); f.bengazi@virgilio.it (F.B.); tmainetti@libero.it (T.M.); caroline7_k@hotmail.com (K.A.A.A.); 2 Department Oral Surgery, Faculty of Dentistry, University of Medical Science of La Habana, La Habana 10400, Cuba; eddytroya1@gmail.com; 3 Department of Oral Biology, University Clinic of Dentistry, Medical University of Vienna, 1090 Vienna, Austria; 4 Independent Researcher, 42121 Reggio Emilia, Italy; grassi@dentistire.it

**Keywords:** alveolar process, alveolar ridge augmentation/methods, bone regeneration, cone-beam computed tomography, guided tissue regeneration, periosteal inhibition, tooth extraction, xenografts

## Abstract

**Background/Obectives:** Alveolar bone resorption after tooth extraction compromises esthetics and implant placement. Conventional alveolar ridge preservation (ARP) relies on grafting. This randomized controlled study evaluated a graft-free, lamina-based approach aimed at preserving ridge morphology by protecting the buccal cortical plate. **Methods:** Forty alveoli were randomly assigned to Guided Alveolar Ridge Preservation (G-ARP) with a subperiosteally positioned cortical lamina (test) or unassisted healing (control; CTRL). Cone-beam computed tomography (CBCT) was performed before extraction and after five months. Vertical and horizontal dimensional changes were statistically compared. **Results:** Healing was uneventful. At five months, the G-ARP group showed a vertical gain of 0.5 mm and a horizontal reduction of 0.2 mm, whereas the CTRL group exhibited a vertical loss of 1.7 mm (*p* < 0.01) and a horizontal loss of 2.7 mm (*p* < 0.001). Effect sizes were large for vertical change and very large for horizontal change (Hedges’ g = 0.95 and 2.19, respectively). Regeneration occurred through native bone formation without grafts. **Conclusions:** Subperiosteal placement of a cortical lamina effectively preserved ridge dimensions after extraction. This graft-free approach may offer technical and biological advantages while supporting new bone regeneration.

## 1. Introduction

Alveolar bone resorption represents a major clinical challenge, as it compromises both the structural integrity of the jaws and the esthetics. Alveolar ridge preservation (ARP) techniques have been introduced to mitigate post-extraction bone loss. While a number of augmentation strategies have demonstrated the ability to stabilize ridge dimensions [1,2,3,4], conclusive evidence regarding their esthetic and prosthodontic benefits remains limited [5,6,7,8].

Tooth extraction, whether due to pathology, endodontic complications, periodontal disease, or trauma, is often performed without measures to preserve the alveolar ridge. Following the removal of a permanent tooth, bone remodeling occurs over several months, with the most pronounced changes observed during the first three months [9]. Significant reductions in alveolar ridge dimensions have been reported, with bucco-lingual width decreasing by up to 50% and vertical height also being affected [9,10,11,12]. A systematic review estimated an average reduction of 3.8 mm in width and 1.24 mm in height within the first six months [13]. The pattern of bone resorption is predictable: the buccal plate is lost first, horizontal loss exceeds vertical loss, and resorption occurs more rapidly in the mandible than in the maxilla [14,15]. A lingual shift of the alveolar crest relative to the original tooth position has also been documented [16].

Over the past two decades, several strategies have been proposed to counteract ridge resorption after tooth extraction. ARP procedures, performed with immediate or delayed implant placement, sought to minimize dimensional changes in the alveolar ridge and surrounding soft tissues by grafting extraction alveoli with different biomaterials or applying a socket-shield technique [17,18,19]. Several systematic reviews have reported that grafting techniques may reduce progressive bone resorption [19,20,21,22].

Systematic reviews and a consensus statement also confirmed that ARP procedures significantly reduced both vertical (up to 1.5–1.7 mm) and horizontal ridge resorption (up to 1.8–2 mm) compared with unassisted alveolar healing [23,24,25,26]. Nevertheless, clinicians frequently select ARP protocols based on individual preference rather than robust evidence. Thus, the true clinical efficacy of specific grafting materials and ARP procedures remains controversial.

More recently, a novel approach has been proposed to enhance alveolar preservation and support the formation of vital new bone [27,28,29,30]. The technique consists of positioning either a high-density polytetrafluoroethylene (d-PTFE) membrane or a cortical lamina between the elevated flap and the buccal bony plate immediately after tooth extraction, thereby isolating the periosteum from the underlying bone. Although initial reports appear promising, a definitive randomized controlled trial has yet to validate this protocol. Hence, the aim of the present randomized clinical trial was to evaluate the effectiveness of placing a cortical lamina between the elevated flap and the buccal bone plate following tooth extraction in maintaining alveolar ridge dimensions. The null hypothesis was that no differences would be observed between test and control sites in terms of horizontal and vertical dimensional changes in the alveolar ridge.

## 2. Materials and Methods

### 2.1. Ethical Statements and Study Design

This was a single-center, prospective, randomized, controlled clinical trial (RCT) with partial blinding that evaluated dimensional changes in the healed alveoli using CBCT following a Guided Alveolar Ridge Preservation (G-ARP) technique (test) applying a cortical lamina on the buccal bony wall compared with unassisted post-extractive alveoli healing (control). The study started in April 2024 and was completed at the beginning of October 2025; it was conducted in full accordance with the ethical principles outlined in the Declaration of Helsinki (2008 revision). Informed consent was obtained from all subjects involved in the study. Ethical approval was obtained from the Research Ethics Committee of the University of Medical Sciences of Havana, Faculty of Dentistry, on 9 April 2024 (Protocol No. 2024/01), prior to patient enrollment.

The trial was retrospectively registered at ClinicalTrials.gov (Identifier: NCT07231874) on 14 November 2025, prior to manuscript submission. The retrospective registration resulted from administrative difficulties during the initial attempt to register the study in the Cuban registry, followed by conflicting guidance regarding the appropriate national registration pathway because the study was supported by an Italian institution. As these administrative issues could not be resolved in a timely manner, the study was ultimately registered at ClinicalTrials.gov. Completion of the registration process was further delayed by temporary administrative and operational constraints in October 2025. No changes to the study protocol, outcomes, or statistical analysis plan were made after study initiation. The reporting of this study adheres to the CONSORT guidelines.

### 2.2. Sample Calculations

A systematic review evaluating dimensional changes in alveolar hard and soft tissues following tooth extraction without intervention reported an average horizontal reduction of 3.8 mm [13]. Applying ARP techniques has been reported to better preserve the alveolar ridge width, reporting a gain of approximately 0.5 mm in width compared with spontaneous healing [28].

Based on a clinically relevant mean horizontal difference of 1.5 mm and a standard deviation of 1.0 mm, the corresponding standardized effect size was Cohen’s d = 1.5. The required sample size for a two-sided, two-sample *t*-test (α = 0.05, allocation ratio 1:1) was estimated in G*Power3.1 (test family: *t*-tests; statistical test: means—difference between two independent means; type of power analysis: a priori). This analysis indicated that 10 participants per group would yield 90% power to detect the hypothesized difference. To accommodate potential dropouts/non-evaluable cases, the target enrollment was increased to 20 per group (total *n* = 40).

### 2.3. Randomization, Allocation Concealment, and Blinding Procedures

The randomization process was carried out electronically by an author (D.B.) who was not involved in the surgical procedures. Treatment allocations were concealed using sealed, opaque, coded envelopes prepared and retained by the same author and opened only after tooth extraction. Patient enrollment and assignment to treatment were performed by an author not involved in the surgical procedures (E.T.B.). All surgeries were performed by G.M., who remained blinded to the treatment allocation until completion of the tooth extraction, after which the allocated procedure was necessarily revealed. Patients were blinded to the treatment assignment, and the examiner responsible for CBCT measurements (T.M.) was fully blinded to group allocation.

### 2.4. Inclusion and Exclusion Criteria

Patients were consecutively assessed for eligibility among those referred for tooth extraction and were included if they were ≥21 years of age and in good general health; had the presence of a hopeless prognosis for a single anterior or premolar tooth in either jaw (molars excluded) requiring extraction and subsequent implant placement; had adequate oral hygiene and agreed to follow the study protocol and scheduled follow-up visits; and provided written informed consent.

Patients were excluded if they were smokers (>10 cigarettes/day) or heavy alcohol consumers; were pregnant or breastfeeding; had uncontrolled systemic conditions (e.g., uncontrolled diabetes, immunocompromised status, bleeding disorders); had been treated in the previous 6 months for active periodontal disease; were under treatment with medications known to interfere with bone or soft tissue healing (e.g., phenytoin, cyclosporine, dihydropyridine calcium channel blockers, anticoagulants, bisphosphonates); had a history of drug abuse or psychological disorders that could compromise adherence; or had insufficient bone volume to allow implant placement after healing, as assessed radiographically.

Cases presenting fistulas, dehiscences, perforations, or apical radiolucencies were not excluded a priori. Eligibility was assessed during the screening phase based on clinical and radiographic criteria and was subsequently confirmed intraoperatively after tooth extraction. Sites presenting minor defects compatible with the predefined surgical protocol were included, whereas sites showing extensive fistulas or buccal defects requiring additional regenerative procedures were excluded.

### 2.5. Biomaterial Used

Cortical lamina (Osteoxenon, Bioteck, Arcugnano, Italy): A natural cortical bone lamina (thickness: 0.5 mm) was used. The lamina was enzymatically deantigenated to remove antigenic components while preserving the native bone collagen matrix. It was partially demineralized to increase flexibility, allowing easy adaptation to the grafted site.

### 2.6. Radiographic Sessions

Cone-beam computed tomographies (CBCTs) were acquired approximately 1 week before tooth extraction and after 5 months, prior to the second-stage surgery for implant placement, using a 3D Hyperion MyRay X5 system (Cefla, Bologna, Italy) with a field of view (FOV) of 10 × 8 cm and a voxel size of 0.3 mm. Exposure parameters were automatically set at 90 kVp and 15 mAs. Coronal images were analyzed at a magnification of ×200 using the dedicated proprietary software provided by the manufacturer (iRYS, Cefla, Bologna, Italy).

During the study, the CBCT system was periodically calibrated to ensure consistent image acquisition and to minimize potential dimensional distortion. Formal intra- and inter-observer agreement statistics were not assessed and represent a limitation of this study.

### 2.7. Clinical Procedures

**Figure 1:**
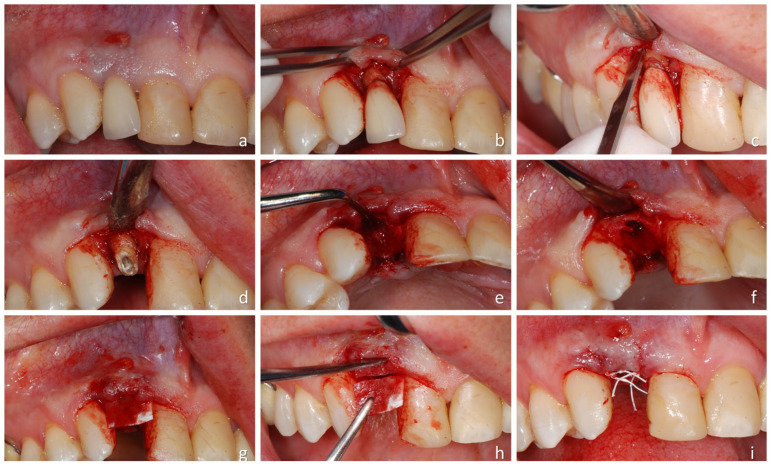
Clinical procedures: (**a**) presence of an apical fistula above tooth 1.2; (**b**) flap elevation; (**c**) detachment of the most coronal fibers using a mini-beaver blade inserted into the sulcus; (**d**) situation after the detachment; (**e**) tooth extraction performed with forceps; (**f**) degranulation of the alveolus; (**g**) creation of a full-thickness tunnel using a periosteal elevator and placement of the cortical lamina between the bone plate and the periosteum; (**h**) adaptation of the excess portion of the lamina to ensure complete coverage by the flap; (**i**) suturing of the surgical site.

Two hours before surgery, the patients were given a single dose of 2 g of amoxicillin and clavulanic acid. Chlorhexidine 0.2% was administered before surgery to rinse the mouth, and paracetamol was administered for immediate postoperative pain control. The region of extraction was selected (Figure 1a) and infiltrated with local anesthesia.

With a surgical blade, an intrasulcular incision was performed, extending mesially and distally. The buccal margin of the gingiva was then carefully elevated with a periosteal elevator to expose the marginal alveolar crest (Figure 1b). A thin, sharp, rounded chisel (mini beaver blade) was introduced into the sulcus along the periodontal ligament, and gentle, small movements were applied to detach the most coronal fibers, taking care to avoid damaging the thin buccal bone plate during extraction (Figure 1c,d). Following tooth removal using forceps, the alveolus was thoroughly degranulated (Figure 1e).

All patients remained enrolled in the study, even in cases where partial damage to the buccal bone plate occurred during extraction. Patients were enrolled sequentially, and treatment allocation (G-ARP or control) was assigned using sealed envelopes prepared by an independent administrator.

#### 2.7.1. G-ARP (Test Group)

Following tooth extraction, a tunnel was created on the buccal aspect of the residual alveolus using a periosteal elevator, positioned beneath the periosteum and soft tissues (Figure 1f). The tunnel extended approximately 8–10 mm in depth, with a lateral spread limited to the mesial and distal papillae to prevent complete detachment of the adjacent gingival tissues. Within this subperiosteal pocket, a cortical bone lamina was carefully shaped and inserted in close adaptation to the denuded buccal alveolar surface (Figure 1g). The excess portion of the lamina was trimmed to allow complete coverage by the flap (Figure 1h). Interrupted sutures were then placed at the mesial and distal papillae, leaving the alveolus intentionally uncovered (Figure 1i). Finally, gentle pressure with saline-soaked gauze was applied for 5 min to stabilize the blood clot.

Lingual cortical laminae were applied in a limited number of cases presenting lingual defects. Fixation pins were used only when adequate primary stability of the lamina could not be achieved by adaptation alone, particularly in the presence of buccal bone defects. The use of fixation pins was considered a technical stabilization measure and not a modification of the surgical protocol.

#### 2.7.2. CTRL Group (Control Group)

After extraction, interrupted sutures were placed at the mesial and distal papillae, leaving the alveolus uncovered. Hemostasis and clot stabilization were obtained by applying sterile saline-soaked gauze under gentle pressure for 5 min.

### 2.8. Postoperative Instructions

Postoperative instructions included the use of a 0.2% chlorhexidine digluconate mouth rinse, three times daily, combined with a modified brushing regimen that avoided the surgical site. Sutures were removed after 14 days, during which the surgeon also performed a clinical control. At the same visit, a dental hygienist provided professional debridement and reinforced oral hygiene instructions; an additional hygienist session was scheduled at 8 weeks.

When required in esthetic areas, patients received an acrylic provisional restoration, such as a Maryland Bridge, placed on the day of surgery. At 5 months following the initial surgery, patients were recalled for implant installation. One week prior to implant placement, a control CBCT was obtained.

### 2.9. Tomographic Examinations

All CBCT measurements were performed by a single calibrated examiner (T.M.) following a predefined measurement protocol. Examiner calibration was achieved through repeated supervised measurement sessions, during which landmark identification and measurement consistency were reviewed by a senior investigator (D.B.). Measurements were checked multiple times, and when discrepancies were identified, the entire measurement sequence was repeated until consistent results were obtained.

Formal inter- and intra-observer agreement statistics were not calculated; however, this supervised calibration and repeated-measurement approach was adopted to reduce measurement variability and enhance internal consistency. All measurements were performed on standardized cross-sectional slices oriented perpendicular to the alveolar ridge to ensure reproducibility.

**Figure 2:**
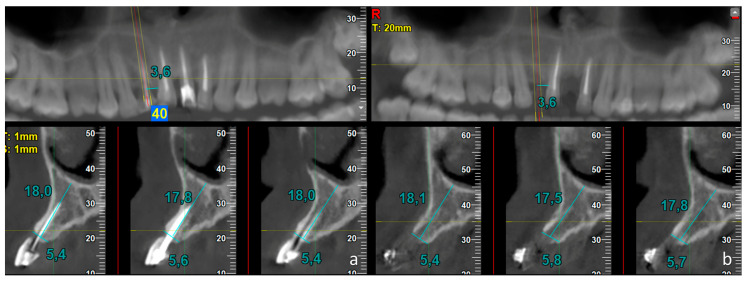
Test site. Cone-beam computed tomographic (CBCT) scans obtained (**a**) before and (**b**) 5 months after tooth extraction. In the panoramic view, the central position of the extracted root was identified. At T1, measurements of crestal width and vertical distance from the nasal floor (blue lines) were performed on the central cross-section (red line) and on the adjacent sections located 1 mm mesially and distally (yellow lines). At T2, the distance from the neighboring tooth was determined on the panoramic view, and the same measurements as at T1 were repeated (horizontal blue lines).

**Figure 3:**
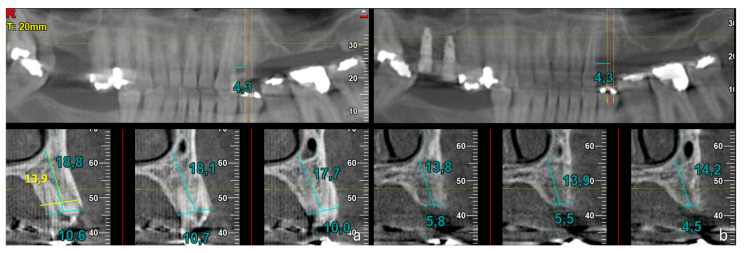
Control site. Cone-beam computed tomographic (CBCT) scans obtained (**a**) before and (**b**) 5 months after tooth extraction. The same measurements described in Figure 2 were performed (blue lines). In addition, in the control group only, the alveolar crest width was measured at the level corresponding to the crestal height observed at T2 (Width_1_). The yellow bucco-lingual line in figure a represents an example of the position where Width_1_ was measured, at the same vertical level as the resorbed bone crest at T2. This additional measurement was performed to allow comparison of ridge width at the same vertical reference level before and after healing.

These measurements were carried out on all CBCT scans obtained before tooth extraction (T1) and after 5 months of healing (T2). At T1 (Figure 2a and Figure 3a), all measurements were carried out on the coronal CBCT section passing through the central axis of the tooth (central aspect) and repeated on the sections located 1 mm mesially and distally to the central one (mesial and distal aspects, respectively).

This approach was adopted to compensate for potential minor discrepancies in the positioning or angulation of the corresponding measurement planes between T1 and T2. The width of the alveolar bone crest and the distance from the line drawn for width measurement to the mandibular base (in the lower jaw) or to the nasal/sinus floor (in the upper jaw) were recorded. Moreover, in the panoramic view, the distance between the central axis of the tooth and the neighboring teeth was also measured.

At T2 (Figure 2b and Figure 3b), the correct position of the central coronal section was identified on the panoramic view using the reference measurements taken at T1. Then, the width of the residual alveolar crest and the distance from this line to the mandibular base or to the nasal/sinus floor were measured. Again, all measurements were carried out in the three sections mentioned above.

An additional exploratory measurement of the alveolar crest width was performed at the control sites only, based on the CBCT scan acquired at T1 (Figure 3a). This measurement was taken at the level corresponding to the height measured at T2, representing the new position of the resorbed alveolar crest. This approach allowed a direct comparison between the resorbed alveolar crest observed at T2 and the original width of the crest at the same level on T1 (Width_1_).

### 2.10. Statistical Analysis

The primary outcome was the horizontal dimensional change measured at the central aspect of the alveolar ridge. All other parameters were regarded as exploratory, although complementary.

Data distribution was assessed using the Shapiro–Wilk test for normality. Depending on the results, either Welch’s *t*-test or the Mann–Whitney U test was applied for group comparisons. Differences between periods were analyzed with either a paired *t*-test or a Wilcoxon test. Statistical significance was set at α = 0.05. Analyses were performed using GraphPad Prism (version 10.6; GraphPad Software, San Diego, CA, USA).

Effect size analyses were prioritized for the primary outcome and its most clinically relevant complementary measurements. The effect size for the central aspect of both vertical and horizontal difference measurements was calculated as Hedges’ g, based on pooled standard deviations and corrected for sample size. The 95% confidence intervals (CI) were computed using standard formulas for independent groups. Values of 0.2, 0.5, and 0.8 were interpreted as small, medium, and large effects, respectively.

Effect size calculations were performed using customized Microsoft Excel (version 365) spreadsheets implementing the corresponding statistical formulas. The effect sizes were interpreted according to the conventional thresholds proposed by Cohen and the extended categorization by Sawilowsky. Accordingly, no adjustment for multiple comparisons was applied to exploratory analyses, which are presented for descriptive and hypothesis-generating purposes.

## 3. Results

**Figure 4:**
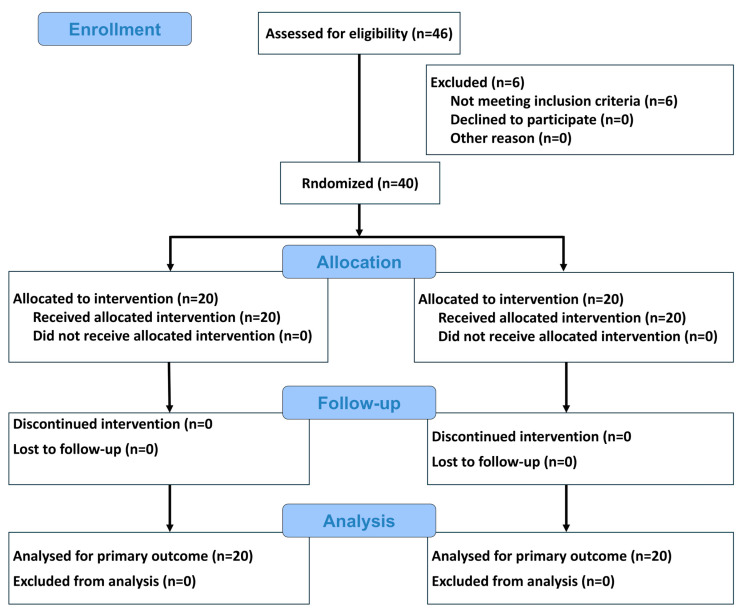
CONSORT Flow Diagram. Flow diagram of the progress through the phases of a randomised trial of two groups (that is, enrolment, intervention allocation, follow-up, and data analysis).

Patient screening, exclusions, and allocation are summarized in Figure 4. Six patients were excluded because they presented extensive fistulas or buccal defects exceeding the limits compatible with the predefined surgical protocol. A total of 40 alveoli were included and allocated to the G-ARP group (*n* = 20) or the control group (*n* = 20).

### 3.1. Clinical Evaluations

While most cases exhibited an intact buccal bone wall, some sites showed dehiscences or fenestrations at the buccal aspect. During healing, no adverse events were recorded. A total of 40 alveoli were treated, with 20 assigned to each group (Table 1).

Maxillary premolar alveoli were the most frequently included in both the G-ARP and CTRL groups (9 and 8, respectively), followed by maxillary incisor alveoli (4 and 5, respectively).

### 3.2. Tomographic Evaluations

All patients underwent CBCT analysis. No statistically significant differences were observed between the test and control groups at T1 for the widths. However, at T2, the widths showed a statistically significant difference in favor of the G-ARP protocol. The reductions in both height and width between the two time points were statistically significant for all variables in the CTRL group, whereas no significant differences were detected in the G-ARP group.

The G-ARP group showed minimal dimensional changes, with width variations ranging from +0.1 mm to −0.2 mm across the three aspects evaluated (Table 2). Vertical alterations were also negligible, ranging from +0.1 mm to +0.5 mm. Conversely, the CTRL group exhibited a marked width reduction ranging from −2.4 mm to −2.7 mm, accompanied by a vertical loss between −1.1 mm and −1.7 mm. Moreover, when comparing the crest width at T2 with that measured at T1 at the same vertical level, the reduction in the CTRL group was slightly greater, showing a loss ranging from −2.8 mm to −3.1 mm.

For the central aspect (primary outcome), the horizontal dimensional change demonstrated a very large effect size (Hedges’ g = 2.19; 95% CI: 1.41–2.97), whereas the vertical dimensional change showed a large effect size in favor of the test group (Hedges’ g = 0.95; 95% CI: 0.30–1.59). While vertical dimensional changes showed a large effect size, these findings should be interpreted as exploratory outcomes, as the study was not powered to test vertical changes as a primary endpoint.

## 4. Discussion

The present randomized controlled study showed that the application of a cortical lamina positioned subperiosteally on the buccal bone plate was associated with minimal dimensional changes in the alveolar ridge following tooth extraction. After five months of healing, both horizontal and vertical alterations were negligible in the test group (ranging from −0.2 to +0.1 mm and +0.1 to +0.5 mm, respectively), whereas significant bone loss occurred at the control sites (ranging from −2.4 to −2.7 mm horizontally and −1.1 to −1.7 mm vertically). These findings indicate that the G-ARP (Guided Alveolar Ridge Preservation) technique successfully achieved its goal of preserving ridge morphology, supporting the working hypothesis that a temporary cortical lamina may help maintain alveolar dimensions during the early phases of healing. Given these significant intergroup differences, the null hypothesis, which stated that no difference would exist between the test and control sites in terms of ridge dimensional changes, was rejected.

It should be noted that the horizontal ridge width at the central aspect was predefined as the primary outcome of the study, whereas vertical dimensional changes were considered exploratory and should be interpreted accordingly.

When compared with previous studies using collagen membranes or particulate grafts, the amount of ridge reduction observed in the present investigation was notably lower. Systematic reviews and a consensus statement have reported average post-extraction losses of 1.5–1.7 mm vertically and 1.8–2.0 mm horizontally, even when bone substitutes and membranes were applied [23,24,25,26]. In contrast, the current findings suggest that lamina-based ridge preservation may achieve similar or superior dimensional stability without graft materials, relying instead on the mechanical and possibly biological properties of the cortical barrier itself.

Notable outcomes have been reported in clinical studies using deproteinized bovine bone mineral combined with collagen membranes, where ridge width losses ranged from 1.0 to 1.8 mm [17,26]. However, the almost complete maintenance of ridge dimensions achieved in the present study highlights the potential benefit of buccal cortical protection rather than alveolar filling alone.

Tooth extraction inevitably leads to the loss of the periodontal apparatus, interrupting the vascular supply and the biological stimuli that previously sustained the surrounding bone. The removal of the tooth also eliminates the mechanical and functional support that maintained the bundle bone and the buccal cortical plate, which often extends beyond the natural contour of the alveolar process. In our study, the control group, in which no intervention was performed, exhibited a reduction in alveolar crest dimensions as a result of these processes. When a cortical lamina was placed, these physiological post-extraction phenomena still occurred; however, the overall dimensions of the alveolar crest remained preserved.

The mechanism underlying ridge preservation associated with subperiosteal placement of a cortical lamina remains hypothetical and is still debated. The concept underlying the G-ARP technique is consistent with the principles of Guided Tissue Regeneration (GTR), first introduced by Sture Nyman, in which selective tissue isolation allows the regeneration of a specific tissue type [31,32,33]. Similarly, the proposed approach aims to promote alveolar bone regeneration by isolating unwanted soft tissues, in analogy with how the periodontal ligament is protected in GTR procedures.

In addition to selective tissue exclusion, successful GTR-based approaches rely on the creation and maintenance of a protected regenerative space. In this context, the cortical lamina may primarily function as a space-maintaining and mechanically protective barrier, counteracting soft-tissue pressure and preventing collapse of the healing compartment, rather than acting as a biologically active graft.

Accordingly, the observed dimensional stability may also be explained by a predominantly mechanical protective effect of the lamina against external forces exerted by the lips, cheeks, and tongue, or by a combination of mechanical and biological factors.

For this reason, we propose the term Guided Alveolar Ridge Preservation (G-ARP) to describe this technique.

In this context, Kivovics et al. [34] introduced the Fibrinogen-Induced Regeneration Sealing Technique (F.I.R.S.T.), which involves the use of a cortical lamina to cover the defect, combined with a fibrin sealant to enhance graft adaptation and to stabilize the collagenic bone shield.

These converging approaches highlight the growing interest in lamina-based ridge preservation and GBR strategies aimed at space maintenance and mechanical stabilization. Nevertheless, despite the consistency of the clinical and radiographic observations reported across different techniques, the precise biological mechanisms underlying their effects remain to be fully clarified.

Within this framework, different explanatory models have been proposed to account for the observed dimensional stability, including periosteal inhibition, altered remodeling dynamics, and combined mechanical–biological interactions.

This conceptual framework is related to the periosteal inhibition hypothesis originally proposed by Nguyen et al. [27], who suggested that separation of the periosteum from the buccal cortical plate using a d-PTFE membrane could limit post-extraction resorption by interfering with periosteal-derived osteoclastic activity. Subsequent clinical reports applying similar principles to cortical laminae have described limited ridge dimensional changes [28,29]. However, these observations are based on small case series and CBCT assessments, and no histological or experimental evidence has demonstrated a causal inhibitory role of the periosteum in alveolar bone resorption.

Importantly, experimental data—including studies conducted by the present research group [35,36]—have shown that physiological remodeling of the buccal alveolar crest may still occur even when the periosteum is physically separated from the underlying bone. These findings suggest that post-extraction resorption is likely governed by multiple biological pathways rather than by periosteal activity alone. Therefore, periosteal inhibition should be regarded as a theoretical explanatory model rather than a demonstrated biological mechanism. At present, no histological or experimental evidence allows attribution of a causal inhibitory role to the periosteum alone, and the observed clinical effects should be interpreted within a multifactorial framework.

A comparable mechanical influence may also be achieved through immediate closure of the alveoli using prosthetic or restorative components, without the placement of grafting materials or barrier membranes. In this context, provisional restorations, immediate implant-supported temporaries, and customized healing abutments have been proposed as non-grafting strategies capable of mechanically sealing the alveolus and stabilizing the early healing environment. Clinical reports have suggested that well-designed provisional prostheses—especially those incorporating ovate pontics, immediate implant-supported temporaries, or customized healing abutments—can contribute to the maintenance of ridge volume. Such prosthetic components help stabilize the blood clot, support peri-implant soft tissues, and reduce collapse of the alveolar contour during early healing by acting as a mechanical seal or “plug” of the alveolus. Although their effect is largely indirect and mediated by soft-tissue tension and mechanical support, some studies have observed slightly reduced ridge resorption both in terms of attenuation of bone remodeling [37,38,39] and mechanical stabilization of ridge contour [40,41,42,43] when immediate provisionalization or customized healing abutments were used. These findings collectively indicate that mechanical stabilization of the periosteal and mucosal envelope may play a meaningful role in mitigating dimensional changes in the alveolar crest during early healing.

A conceptually related but biologically distinct approach is represented by the socket-shield technique, first described by Markus Hürzeler et al., which aims to preserve a buccal root fragment in situ in order to maintain the contour of the alveolar ridge [44].

Unlike socket-shield procedures, the present G-ARP approach does not rely on the preservation of dental tissues but rather on the creation of a mechanically protected and space-maintained environment, thereby reducing the risk of technique-sensitive biological assumptions.

In the present experiment, no protection was provided to the entrance of the alveoli, nor was any graft material applied. Within the limits of this study, the results suggest that socket filling may not be necessary to achieve short-term dimensional preservation when a subperiosteal cortical barrier is used. This represents an important advantage from a technical and biological standpoint, as well as from an economic perspective. The procedure required only the preparation of a subperiosteal tunnel on the buccal aspect, followed by the placement of a cortical lamina beneath the full-thickness flap. Fixation pins were employed only in a few cases where the conditions of the buccal bone plate or soft tissues did not provide sufficient primary stability of the lamina. The biological advantage of this approach lies in the regeneration of new bone without residual graft material that might interfere with subsequent osseointegration of implants [45].

Exploratory measurements of the alveolar crest width on the T1 CBCT, at the level corresponding to the T2 height line, enabled comparison between the healed (resorbed) crest and the original alveolar dimensions at the same vertical level. This approach minimized potential biases that could result from evaluating width changes at different heights, which might otherwise underestimate the true extent of post-extraction resorption. As a result, the procedure provided a more consistent and accurate assessment of the dimensional modifications that occurred during healing and revealed clearer differences between the test and control groups. Although from a clinical standpoint, the most relevant outcome remains the residual ridge available for implant placement, this analytical strategy improved the internal validity and interpretative strength of the present findings.

From a clinical perspective, early preservation of ridge dimensions represents a critical determinant for simplifying subsequent implant placement and minimizing the need for additional augmentation procedures. Although long-term implant outcomes were not evaluated, the nearly complete maintenance of alveolar width and height observed at five months suggests that the G-ARP approach may facilitate implant placement in prosthetically driven positions, potentially reducing surgical complexity, treatment time, and patient morbidity.

In routine clinical practice, ridge collapse frequently necessitates secondary regenerative interventions, increasing costs, morbidity, and the overall treatment burden. Therefore, a technique capable of preserving ridge morphology during the early healing phase, even without graft materials, may have relevant clinical implications by enabling more predictable implant placement strategies. However, the clinical benefit may be less predictable in molar sites, in sockets with extensive buccal defects, or in protocols involving immediate implant placement, where different biomechanical and biological conditions apply.

While the present results cannot be extrapolated to long-term implant success or prosthetic outcomes, they provide controlled, hypothesis-driven evidence supporting the concept that early mechanical protection of the buccal plate can meaningfully influence post-extraction dimensional remodeling. These findings establish a biologically and clinically plausible rationale for further long-term clinical trials integrating implant placement and prosthetic evaluation.

This study presents several limitations that should be considered when interpreting the findings. First, the CBCT-based measurements may have been affected by artifacts, particularly at T1, due to the presence of teeth, metallic posts, endodontic fillings, or prosthetic restorations. These elements could have caused local distortions or scattering, potentially influencing the accuracy of the baseline dimensional values. Moreover, poorly defined bone crests may have introduced additional measurement errors. The resorption of the buccal bone crest at the control sites may also have altered the direction of the reference line used to assess vertical changes, shifting it slightly toward the palatal aspect and thereby increasing the apparent vertical loss. Although every effort was made to standardize patient positioning and image calibration, such variability cannot be completely ruled out.

Second, the sample size was relatively limited, and the observation period was restricted to five months, allowing only a short-term evaluation of ridge stability. Therefore, the present findings should be interpreted as early healing outcomes and cannot be extrapolated to medium- or long-term functional or restorative results. Although this time point is clinically relevant for assessing early dimensional stability prior to implant placement, it does not allow any conclusions regarding medium- or long-term ridge stability, implant survival, or prosthetic success. Therefore, the observed preservation of ridge dimensions should be interpreted as an early post-extraction effect rather than as evidence of sustained functional or restorative benefit.

Furthermore, the analysis was radiographic rather than histologic, and therefore, the true biological quality of the regenerated bone could not be assessed. In addition, although CBCT measurements were performed following a supervised calibration process with repeated measurements, formal inter- and intra-observer agreement statistics were not calculated and should be considered a limitation of the present study.

From an external validity perspective, the present study is also limited by the anatomical distribution of the treated sites. Maxillary anterior and premolar regions were predominant, and molar sites were excluded. Therefore, the present findings primarily apply to anterior and premolar extraction sites and to clinical scenarios involving delayed implant placement. Caution is required when extrapolating these results to molar regions or to protocols involving immediate implant placement.

Given the surgical nature of the intervention, full double blinding was not feasible, as the surgeon became aware of the allocated procedure after tooth extraction; however, patient blinding and blinded outcome assessment were maintained.

An additional limitation of this study is the retrospective registration of the clinical trial. Although ethical approval was obtained prior to patient enrollment and the study was conducted prospectively according to a predefined protocol, retrospective registration may be associated with an increased risk of reporting bias and should be considered when interpreting the findings. However, no changes to the study protocol, outcomes, or statistical analysis plan were made after study initiation, and all predefined outcomes are transparently reported. Moreover, the assessed outcomes are those commonly adopted in studies on alveolar ridge preservation; the between-group differences were large, and the sample size was more than adequate for the primary endpoint. Therefore, although this limitation must be acknowledged transparently, it is unlikely to have materially affected the main interpretation of the findings.

Despite these limitations, the present findings provide valuable preliminary evidence supporting the potential of a graft-free, lamina-based approach for preserving alveolar ridge dimensions after tooth extraction. The magnitude of the effect sizes, very large for horizontal maintenance (2.19) and large for vertical preservation (0.95), suggests that the lamina may exert a clinically meaningful protective influence on the alveolar contour, further reinforcing the rationale for exploring this graft-free approach in future controlled trials. From a clinical standpoint, the observed short-term preservation of ridge morphology may simplify subsequent implant placement and reduce the need for additional regenerative procedures, although long-term clinical trials remain necessary to confirm these potential benefits.

## 5. Conclusions

Within the limits of this pilot RCT study, the subperiosteal placement of a cortical lamina on the buccal aspect appears to be a promising and minimally invasive approach for limiting short-term alveolar ridge dimensional changes after tooth extraction, which could be advantageous during the pre-implant healing phase. The approach may offer technical advantages, as it requires no graft material and involves a simple surgical procedure with limited morbidity.

From a biological standpoint, this graft-free protocol is associated with ridge preservation without the presence of residual biomaterial particles, which could be advantageous for subsequent implant placement. The maintenance of ridge morphology observed in this study is likely related to a mechanical protective effect of the cortical lamina on the buccal plate.

In addition, the economic implications of a graft-free protocol may increase the clinical attractiveness of this technique. Nevertheless, the underlying mechanism—whether primarily mechanical or partly biologically modulated—remains to be fully elucidated.

However, these findings are based on a five-month pre-implant placement radiographic evaluation and cannot be extrapolated to medium- or long-term clinical, functional, or prosthetic outcomes. Further studies with longer follow-up periods are necessary to confirm the stability and clinical relevance of the observed dimensional preservation.

Future investigations involving larger samples, extended follow-up periods, and histologic evaluation are required to confirm these preliminary findings and to clarify the biological response associated with lamina-based ridge preservation.

## Figures and Tables

**Table 1:** Demographic data and distribution of extraction sites in the test (G-ARP) and control groups. Age is expressed as mean (standard deviation). U = upper; L = lower.

	Male	Female	Age	Incisors	Canines	Premolars
G-ARP	11	9	61.1 (11.5)	4 U, 0 L	2 U, 1 L	9 U, 4 L
CTRL	10	10	61.8 (7.6)	5 U, 2 L	2 U, 1 L	8 U, 2 L

**Table 2:** Vertical and horizontal dimensions of the alveolar crest in millimeters. * *p* < 0.05, ** *p* < 0.01, *** *p* < 0.001: test vs. control; ^†^ *p* < 0.05, ^††^ *p* < 0.01, ^†††^ *p* < 0.001: T1 vs. T2 within the same group.

	Height			Width		
Pre-extraction	Mesial	Central	Distal	Mesial	Central	Distal
G-ARP	17.8 ± 6.9	17.1 ± 7.0	17.6 ± 6.6	7.8 ± 1.4	8.1 ± 1.3	8.0 ± 1.5
CTRL	19.0 ± 7.4 ^†^	19.2 ± 7.4	18.5 ± 8.0 ^†^	8.1 ± 1.5	8.3 ± 1.6	8.2 ± 1.7
Width_1_				8.4 ± 1.8	8.5 ± 1.8	8.5 ± 1.9
Post-extraction						
G-ARP	17.8 ± 6.4	17.6 ± 6.5	17.7 ± 6.6	7.9 ± 1.5 ***	8.0 ± 1.5 ***	7.9 ± 1.7 ***
CTRL	17.9 ± 7.0 ^†^	17.5 ± 6.9 ^††^	17.4 ± 7.4 ^†^	5.6 ± 1.4 ***^,†††^	5.6 ± 1.5 ***^,†††^	5.5 ± 1.8 ***^,†††^
Difference						
G-ARP	0.1 ± 1.4 *	0.5 ± 1.9 **	0.2 ± 1.6	0.1 ± 1.1 ***	−0.2 ± 0.9 ***	−0.2 ± 0.8 ***
CTRL	−1.2 ± 1.9 *	−1.7 ± 2.6 **	−1.1 ± 2.4	−2.4 ± 1.3 ***	−2.7 ± 1.3 ***	−2.7 ± 1.7 ***
Difference at level Width_1_				−2.8 ± 1.4 ***	−2.9 ± 1.3 ***	−3.1 ± 1.6 ***

## Data Availability

The raw data supporting the conclusions of this article will be made available by the authors on request.
